# Oleic acid ameliorates palmitic acid-induced ER stress and inflammation markers in naive and cerulein-treated exocrine pancreas cells

**DOI:** 10.1042/BSR20190054

**Published:** 2019-05-14

**Authors:** Karin Ben-Dror, Ruth Birk

**Affiliations:** Department of Nutrition, Health Sciences School, Ariel University, Israel

**Keywords:** acinar cells, ER stress, monounsaturated fatty acids, pancreas, saturated fatty acids

## Abstract

Dietary fat overload (typical to obesity) increases the risk of pancreatic pathologies through mechanisms yet to be defined. We previously showed that saturated dietary fat induces pancreatic acinar lipotoxicity and cellular stress. The endoplasmic reticulum (ER) of exocrine pancreas cells is highly developed and thus predisposed to stress. We studied the combination of saturated and unsaturated FAs in metabolic and pancreatitis like cerulein (CER)-induced stress states on cellular ER stress.

Exocrine pancreas AR42J and rat primary exocrine acinar cells underwent acute (24 h) challenge with different FAs (saturated, monounsaturated) at different concentrations (250 and 500 µM) and in combination with acute CER-induced stress, and were analyzed for fat accumulation, ER stress unfolded protein response (UPR) and immune and enzyme markers. Acute exposure of AR42J and pancreatic acinar cells to different FAs and their combinations increased triglyceride accumulation. Palmitic acid significantly dose-dependently enhanced the UPR, immune factors and pancreatic lipase (PL) levels, as demonstrated by XBP1 splicing and elevation in UPR transcripts and protein levels (*Xbp1,Atf6, Atf4, Chop, Tnfα, Tgfβ* and *Il-6*). Exposure to high palmitic levels in a CER-induced stress state synergistically increased ER stress and inflammation marker levels. Exposure to oleic acid did not induce ER stress and PL levels and significantly decreased immune factors in an acute CER-induced stress state. Combination of oleic and palmitic acids significantly reduced the palmitic-induced ER stress, but did not affect the immune factor response. We show that combination of monounsaturated and saturated FAs protects from exocrine pancreatic cellular ER stress in both metabolic and CER-induced stress.

## Introduction

Obesity and high fat diet (HFD) are risk factors in the etiology of pancreatitis and pancreatic cancer [1]. Evidence suggests that excess of free fatty acids (FFA) is a factor linking obesity and related pathological conditions. Exocrine pancreas acinar cells have the highest rate of protein synthesis of any mammalian organ and contain a highly developed endoplasmic reticulum (ER) system [2,3]. Thus, ER homeostasis is particularly essential in acinar cells. Perturbations in ER function triggers ER stress and the unfolded protein response (UPR), tightly controlled intracellular signal transduction pathways programmed to re-establish protein homeostasis by improving protein folding, reducing protein synthesis and eliminating misfolded protein aggregates [4,5]. The UPR includes three major simultaneous response pathways, initiated by the ER chaperone BiP (also known as Glucose-related protein 78; GRP78): inositol-requiring enzyme-1 (IRE1), activating transcription factor-6 (ATF6) and protein kinase RNA-activated-like ER kinase (PERK). The importance of UPR in exocrine acinar cells has been demonstrated in UPR X-box binding protein 1 (XBP1) null mutant mice, which have an abnormal exocrine pancreas with poorly developed ER, decreased expression of ER chaperones and marked cellular apoptosis [6–8]. Under conditions of severe and/or prolonged ER stress, when the adaptive/protective UPR responses are enhanced or dysfunctional, the UPR can also activate cell death programs.

Pancreatic acinar cells are highly susceptible to endogenic and exogenic ER perturbations, such as dietary factors, alcohol, smoking, altered metabolism, xenobiotics and reactive oxygen species. In fact, ER stress has been demonstrated to occur in pancreatitis [9]. Recently, ethanol exposure has been shown to cause pancreatitis through an ER stress–related mechanism [10,11]. Conversely, activation of the ER-induced UPR can attenuate pancreatitis and is necessary for the maintenance of pancreatic homeostasis [6,12]. It is yet unknown whether ER stress and pancreatic pathologies caused by other environmental insults are mediated by an insufficiently robust UPR.

Overload of FAs, typical of obesity, is a risk factor for many chronic diseases, including diabetes, pancreatitis and pancreatic cancer [13]. Increased FA levels result in accumulation of fat in non-adipose tissues and a lipotoxic effect, as was demonstrated in many cell types and chronic diseases [13,14]. ER stress plays an important role in the capacity of cells to deal with nutrient flow and fluctuations. Some nutrients, such as saturated fats, have been shown to induce ER stress. In fact, HFD is a well-known inducer of UPR and pathological ER stress signaling [15]. Mice fed a HFD, as well as ob/ob obese mice, demonstrate elevated phosphorylated PERK and IRE1α and JNK activation in both adipose tissue and liver [16,17]. ER stress induced by HFD has also been associated with insulin resistance mechanisms and type 2 diabetes.

Several studies have shown that different types of FAs have distinct effects on cellular stress, where saturated FAs have significant toxic effects compared with unsaturated FA, although the mechanism of this effect is unknown For instance, it has been demonstrated in hepatocytes, β-cell lines, tumor cells, brain cells and muscle cells that exposure to saturated FAs results in ER stress, activation of UPR stress sensors and a pro-inflammatory state [17,18–23]. However, ER stress in pancreatic acinar cells is not well studied. We have shown that pancreatic acinar cells challenged with saturated FAs develop cellular lipotoxicity [24]. Furthermore, recently, we have shown that saturated palmitic acid (PAL) induces significant ER stress in acinar cells while monounsaturated oleic acid (OL) does not [25]. Palmitic and OLs are the most abundant FAs in the body, both reflecting adipose tissue reservoirs and dietary fat consumption [26]. In obesity, there is a sharp elevation in FFA, especially PAL and OL. FAs are a known inducer of CCK release [27,28]. Thus, high levels of both circulating FFAs and CCK are common in pancreas pathologies.

However, whether combination of FAs, with or without cerulein (CER)-induced pancreatitis stress, induces ER stress in pancreatic acinar cells has not been determined. CER is a CCK synthetic analog, applied in high doses to establish acute pancreatitis both *in vivo* and *in vitro* models [29,30].

In our current research, we explored the effects of the combination of both FAs (PAL and OL, at concentrations typical of normal and obese states) on ER and immune stress markers combined with CER-induced stress (mirroring endogenous cellular exocrine pancreas pancreatitis processes) in exocrine *in-vitro* AR42J and *ex-vivo* primary acinar cell models. Our hypothesis was that the combination of FAs (monounsaturated and saturated) can potentially alleviate the cellular stress which contributes to pancreatic pathologies. Based on this study, dietary recommendations for specific combinations of FAs could be incorporated in the treatment regimens for prevention and/or amelioration of pancreas pathologies.

## Experimental

### Exocrine pancreas models

#### Cell culture

Rat pancreatoma AR42J cells (American Type Culture Collection, MD, USA) were maintained as a sub-confluent monolayer culture in Dulbecco’s Eagle’s medium (Rhenium, Jerusalem, Israel) containing 10% (v/v) fetal calf serum (Biological Industries, Beit Haemek, Israel) and 1% (v/v) penicillin–streptomycin. Cells were grown in 5% CO_2_ at 37°C. Dexamethasone (100 nM; Sigma-Aldrich, Rehovot, Israel) was added to the cells for 48 h before the experiments to induce cell differentiation. Tunicamycin (TM, 5 µg/ml) was used as appositive ER stress inducer (Sigma-Aldrich, Rehovot, Israel). CER (10 nmol/l) (Sigma-Aldrich, Rehovot, Israel) was added as stress inducer.

#### Primary pancreatic acinar cells

Sprague–Dawley rats were purchased from Harlan (Rehovot, Israel) and housed and treated according to the institutional ethics guidelines (ethics approval #IL-63-01-15). Rat primary cells were isolated and cultured as previously described by Rickmann et al. [31]. Cells were grown at 37°C under 5% (v/v) CO_2_ atmosphere. Twenty-four hours after isolation, acini were transferred into a new culture dish.

#### FFA load

Fatty acid supplemented medium was prepared with slight modification of the protocol of Spector [32]. Briefly, Palmitic acid (PAL) and OL (Sigma-Aldrich, Rehovot, Israel) were dissolved in ethanol, and then gently mixed until completely dissolved, after which the clear fatty acids solution was complexed with fatty acid free BSA (Roche) at 10:1 FA to BSA ratio. The complex fatty acid solution was added to the serum-containing cell culture medium to obtain the indicated final FFA concentration. The control untreated cells received the same vehicle solution without the FA. The FA concentrations are typical to normal and obese plasma concentration of FFAs (250 and 500 μmol/l) [33].

### Fat accumulation: oil red O staining

Oil red *O* staining was performed using slight modifications of the protocol of Koopman et al. [34]. Briefly, cells were grown on cover glass slides and were subject to different FA treatments as indicated. After removal of medium, cells were washed with PBS solution (×2) and chemically fixed using 20% formaldehyde solution for 20 min followed by three rinses in deionized water for 30 s. Subsequently, the glass slides were incubated in oil red *O* solution (Sigma, Israel) for 30 min and washed with deionized water. Cells were examined and photographed by light microscopy (Nikon ECLIPSE TE2000-U). There were five coverslips in each group and time point, with an average of 20 fields examined in each slide. Oil red O staining was quantified by Isopropyl alcohol extraction and analyzed by spectrophotometer (510 nm).

### RNA isolation and cDNA synthesis

Total RNA was isolated from cultures of AR42J cells using TRIzol Reagent (Invitrogen, Rhenium, Modi’in, Israel), based on Chomczynski and Sacchi protocol [35]. RNA integrity was tested by agarose gel electrophoresis (0·8% w/v) with ethidium bromide staining. RNA was quantitated by UV absorption at 260 nm using a spectrophotometer (NanoDrop ND-1000 UV-Vis; NanoDrop Technologies, DE, USA). Total RNA was quantified by UV absorption and reverse transcribed into cDNA using SuperScript II reverse transcriptase and oligo-dT primers (Invitrogen, Rhenium, Modi’in, Israel). cDNA was further analyzed by real-time PCR.

### Quantitative RT-PCR

Transcript levels were determined by quantitative PCR (QPCR) using SYBR^®^ Green PCR Master Mix (Life Technologies, Rhenium, Modi’in, Israel). (https://www.sigmaaldrich.com/technical-documents/protocols/biology/sybr-green-qpcr.html). Gene specific primers were designed using the Primer Express Software (Life Technologies, Rhenium, Modi’in, Israel). The qPCR primer pairs were designed across exons to avoid false positive signals from potentially contaminating genomic DNA. Primer and cDNA concentrations were optimized (including melt curve analyses). The internal reference was ROX. Primer sequences are available upon request. Commercial software (Applied Biosystems, CA, USA) was used to calculate ΔΔCt (2^−(Ct^_Target gene_^− Ct^_control gene_) relative expression values for all the genes studied, normalized to control.

### Western blot analysis

Proteins were extracted using RIPA lysis buffer (15 mM Tris-HCl, 1%, Triton X-100, 0.1%, SDS, 167 mM NaCl, 0.5% sodium deoxycholatic acid), with protease inhibitors cocktail (Sigma-Aldrich, Rehovot, Israel). Total protein (30 μg) was loaded. SDS/PAGE, protein transfer and Western blotting were performed using standard laboratory techniques [36]. Primary antibodies were: goat anti–XBP1 (1–2 µg/ml, Abcam, Zotal, Tel-Aviv, Israel), mouse anti-Hsp90 (1:3000, BD Biosciences), mouse anti-ATF6 (1–5 µg/ml Abcam, Zotal, Tel-Aviv, Israel), rabbit anti- TNF–α (0.1 - 0.2 µg/ml, Abcam, Zotal, Tel-Aviv, Israel), mouse anti-IL6 (0.2–0.4 µg/ml, Abcam, Zotal, Tel-Aviv, Israel), rabbit anti- TGFβ (0.1–1 µg/ml, Abcam, Zotal, Tel-Aviv, Israel), goat anti PL (1:100–1000, Santa Cruz Biotechnology, Almog, Shoham, Israel). The following secondary antibodies were used: goat anti mouse IgG (1:5000, Millipore, Mercury, Rosh Haayin, Israel) and goat anti rabbit IgG (1:3000 Abcam, Zotal, Tel-Aviv, Israel). Luminata-crescendo western HRP substrate chemiluminescence (Millipor, Mercury, Rosh Haayin, Israel) was used to develop the blots. Densitometry analyses of immunoblots were performed using imageQuantTL software (GE life sciences, NJ, USA). All proteins were quantified relative to house-keeping gene.

### *Xbp1* splicing

Xbp1 splicing protocol was done according to Casas-Tinto et al. [37]. Briefly, following PCR amplification of Xbp1 fragment, restriction analysis with Pst1 enzyme (Thermo) was done and products were run on 0.8% agar-gel. The unspliced (non-stressed) variant of Xbp1 (289 bp) results in two fragments of 173 and 116 bp (Xbp1u), whereas the spliced (stressed) form is 266 bp (Xbp1s). All agar-gel bands were quantified using Image J program and the ratio between all spliced and unspliced bands was calculated.

### Immunocytochemistry

Immunocytochemistry was performed as follows: AR42J cells were grown and differentiated on cover slips and were subject to treatments as indicated. After fixation with 4% paraformaldehyde/PBS for 20 min, the cells were permeabilized with Fmbilztzin solution (2.5% Triton/PBS) and incubated in blocking solution (1% Triton, 3% iNGS/PBS) for 1 h followed by overnight incubation with primary rabbit antibodies against XBP1 (Abcam, Israel), detected using goat anti rabbit-IgG coupled to Alexa555 (Abcam, Israel). Nuclear labeling was performed with DAPI (Sigma, Israel). Immunofluorescence were visualized with a LSM 700 Zeiss confocal microscope.

### Statistical analyses

Data are reported as means ± S.E.M. Differences between the means values were tested for statistical significance by ANOVA.

## Results

### FA overload induces accumulation of fat in exocrine pancreas AR42J cells

**Figure 1:**
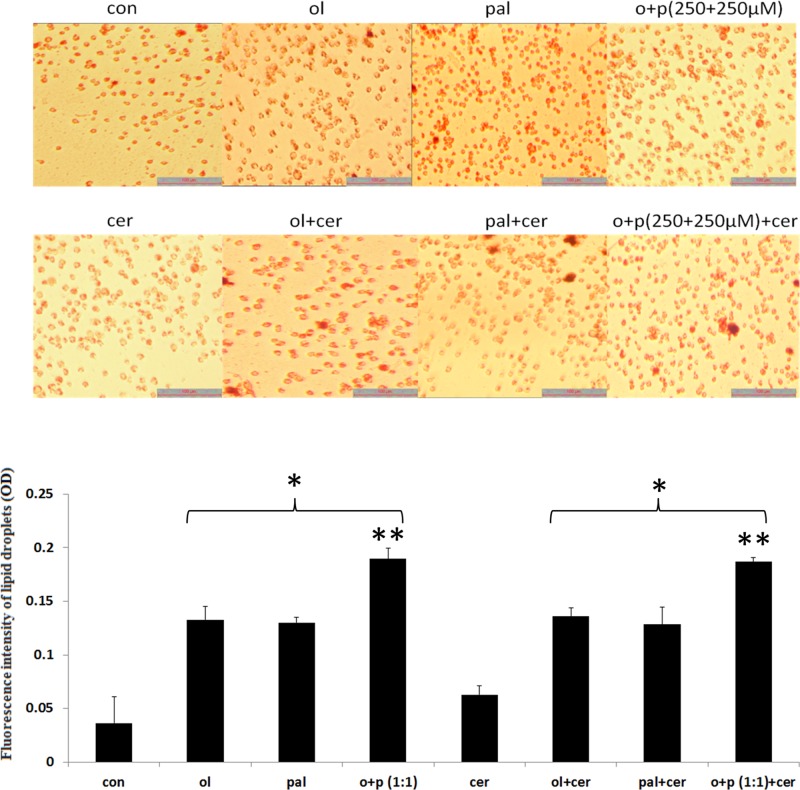
Overload of FA-induced accumulation of fat in exocrine pancreas AR42J cells Quantification of fat accumulation following exposure to FAs and FA combination with or without CER. AR42J cells were grown and differentiated as described in the Experimental section. Differentiated cells were treated with the indicated FAs (250 µM), FAs combination (500 µM) and with CER in 5% FCS medium for 24 h. Subsequently, cells were subjected to oil red O staining followed by image capturing in bright-field microscopy at ×20 magnification. Ruler indicates 100 µM. Quantification analysis of fat accumulation was carried out. Results shown are representative of two independent experiments. Values are given as mean ± SEM. * Asterisk indicates a statistically significant difference (*P*<0.05) versus CON; ** Asterisks indicate a statistically significant difference (*P*<0.05) versus all other treatments.

Exocrine pancreas acinar AR42J cells were treated with: PAL (250 µM), OL (250 µM), FA combination (OL+PAL) with or without CER (10 nmol/l). Treatment with PAL, OL, PAL + OL, OL + CER, PAL + CER and OL + PAL + CER significantly (*P*<0.05) increased fat accumulation compared with control untreated cells. Cells treated with combination of FAs with or without CER showed significant (*P*<0.05) increase in fat accumulation compared with cells treated with only one FA or one FA in combination with CER. Cells treated with CER alone did not significantly differ in fat accumulation from untreated control cells (Figure 1).

### FA acute overload induces ER stress: *Xbp1* splicing in exocrine pancreas primary cells

**Figure 2:**
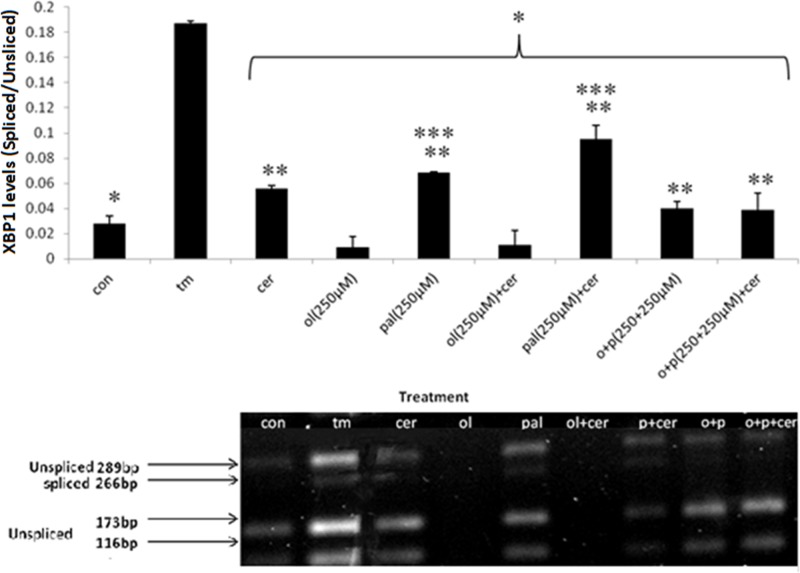
Acute overload of FA (250 µM)-induced ER stress: *Xbp1* splicing in exocrine pancreas primary cells Primary exocrine pancreas acinar cells were treated with different FAs (as indicated). Following treatments, mRNA was isolated and cDNA was synthesized. Xbp1 splicing was analyzed (see Experimental). The unspliced (non-stressed) variant of Xbp1 (289 bp) results in two fragments of 173 and 116 bp (Xbp1u), whereas the spliced (stressed) form is 266 bp (Xbp1s). The photograph is one representative result out of three independent experiments. The results were analyzed by imageJ program and are presented as spliced/unspliced ratio. Results are shown as means ± SEM. * Asterisk indicates a statistically significant difference (*P*<0.05) versus TM; ** Asterisks indicate a statistically significant difference (*P*<0.05) versus CON; *** Asterisks indicate a statistically significant difference (*P*<0.05) versus CER.

**Figure 3:**
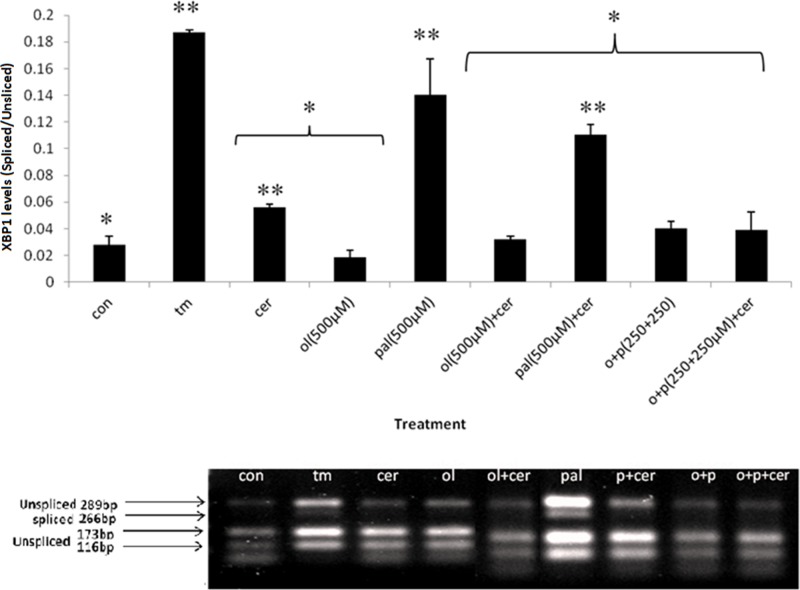
Acute overload of FA (500 μM)-induced ER stress: *Xbp1* splicing in exocrine pancreas primary cells Primary exocrine pancreas acinar cells were treated with different FAs (as indicated) for 24 h. Following treatments, mRNA was isolated and cDNA was synthesized. Xbp1 splicing was analyzed (see Experimental). The unspliced (non-stressed) variant of Xbp1 (289 bp) results in two fragments of 173 and 116 bp (Xbp1u), whereas the spliced (stressed) form is 266 bp (Xbp1s). The photograph is one representative result out of three independent experiments. The results were analyzed by densitometry and are presented as spliced/unspliced ratio. Results are shown as means ± SEM^.^ * Asterisk indicates a statistically significant difference (*P*<0.05) versus TM; ** Asterisks indicate a statistically significant difference (*P*<0.05) versus CON.

*Xbp1* splicing at the mRNA level is a hallmark of UPR activation. We studied the effect of treatment with the FAs OL and PAL (250 µM) and their combination (1:1 ratio), with or without induction of stress (CER), on splicing of *Xbp1* mRNA (*sXbp1*) in primary cells. Following exposure to TM (chemical ER stress inducer), *sXbp1* levels were significantly increased 6-fold as compared with control and to all other treatments, indicating that ER stress was induced in these cells as expected. PAL and PAL+CER treatments significantly increased *sXbp1* levels compared with untreated control cells and to treatment with CER alone. Treatment of cells with PAL, PAL+OL, PAL+CER or CER resulted in significant increase in *sXbp1* levels compared with these treatments without PAL and to control untreated cells, **indicating that the presence of PAL, with or without induction of stress (CER), significantly elevates *sXbp1* levels** (Figure 2). The addition of OL to PAL (with or without CER) significantly reduced *sXbp1* levels. Exposure of primary cells to high levels of OL or PAL (500 μM) significantly elevated *sXbp1* levels compared with lower levels of OL and PAL (250 μM). Cells treated with high levels of PAL (500 μM), with or without CER, demonstrated levels of *sXbp1* comparable with those of cells treated with TM. Exposure of cells to treatment with high OL + CER significantly reduced *sXbp1* compared with treatment with CER alone, attaining levels similar to those of untreated control cells (Figure 3).

These results indicate that *sXbp1* levels are significantly elevated by PAL with further activation with CER, while OL (with or without CER) alleviates this induction of ER stress as indicated by reduction in *Xbp1* splicing.

### FA acute overload and CER-induced stress affect UPR transcript levels

First, we studied whether treatment of cells with low or high FA levels (250 and 500 µM, respectively) affects expression of UPR, immune (*Xbp1, Atf6, Atf4, Chop, Tnfα, Tgfβ* and *Il-6*) and enzyme pancreatic lipase (*pl*) genes. Treatment with high PAL levels resulted in significant (*P*<0.05) elevation in expression of all those genes, as compared with cells treated with low levels (250 µM) of PAL and OL or high levels of OL and control. Treatment with high OL levels was comparable with control untreated cells and to low OL levels; however, treatment with low levels of OL had no effect in comparison with control untreated cells (Supplementary Material, Figure 1). Taken together, these results indicate that the level of FA is a factor affecting UPR, immune and PL levels, especially regarding PAL. Due to the significant results regarding high FA levels, we further studied exposure to high levels of FA with or without CER-induced stress conditions.

**Figure 4:**
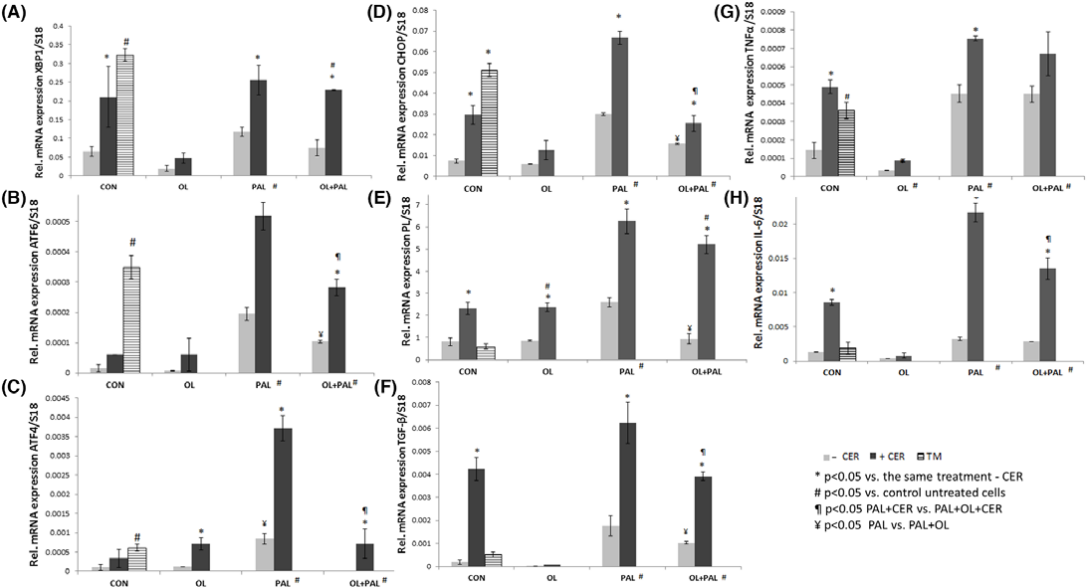
A-H: ER stress, immune and pancreatic enzyme transcripts levels – in the presence of FAs, their combination and CER stress Primary exocrine pancreas acinar cells were isolated and exposed to different FAs, their combination without or with CER (500 µM, FA – grey columns, FA+CER – black columns, TM (positive control) – hatched column) for 24 h as described in Experimental. Cells total RNA was isolated followed by cDNA synthesis and QPCR analysis. Statistical analysis of QPCR was carried out using the (2-ΔΔCt) method, normalized to S18 housekeeping gene and expressed as fold change. (**A**) *Xbp1* (**B**) *Atf6* (**C**) *Atf4* (**D**) *Chop* (**E**) *Pl* (**F**) *Tgfβ* (**G**) *Tnfά* (**H**) *Il6*. Results are expressed as means ± SEM (*n* = 3). Columns with a superscript are significantly different (*P*<0.05). Asterisk * represents significant statistical difference between the treatment versus the same treatment without CER; hash sign # represents significant statistical difference between the treatment and the control untreated cells (# near horizontal treatment title indicates that both columns are different from control). ¥ represent significant statistical difference between OL+PAL versus PAL. ¶ represent significant statistical difference between OL+PAL+CER versus PAL+CER.

CER treatment resulted in significant elevation in *Xbp1* transcripts, reaching levels comparable with those following TM treatment. Treatment with CER+PAL, with or without addition of OL, significantly elevated *Xbp1* transcript levels compared with cells treated with PAL alone, PAL+OL and to control untreated cells (Figure 4A). It should be noted that both absolute levels of fat and type of fat induced changes in *Xbp1* transcript levels. Combination of OL with CER, with high PAL, or with CER+PAL, reduced *Xbp1* transcript levels below the levels seen with these treatments without OL. Treatment with PAL, PAL+OL, or both with CER resulted in significant (*P*<0.05) elevation in *Atf6* transcript levels compared with levels of untreated control cells. Treatment of cells with high levels of OL, with or without CER, had no effect in comparison with untreated cells. Treatment with high levels of CER+PAL and high levels of CER+PAL+OL resulted in significant (*P*<0.05) elevation in *Atf6* transcript levels compared with cells treated with OL with or without CER and control untreated cells. Moreover, CER+PAL (high) treatment showed significant (*P*<0.05) and highest elevation in *Atf6* transcript levels compared with all treatments (Figure 4B). Addition of OL to either PAL or PAL+CER treatments significantly reduced *Atf6* transcript levels compared with these treatments without OL.

Treatment of cells with OL+PAL (500 µM) did not induce detectable *Atf4* transcript levels. Nevertheless, treatments of cells with PAL without or with CER and OL+CER showed significant (*P*<0.05) elevation in *Atf4* transcript levels compared with control cells. In addition, cells treated with CER (in conjunction with OL, PAL and OL+PAL) showed significant (*P*<0.05) elevation in *Atf4* transcript levels compared with these treatments without CER. PAL, OL+PAL and OL+CER treatments significantly (*P*<0.05) elevated *Atf4* transcript levels compared with controls cells. Addition of OL to PAL with or without CER significantly reduced *Atf4* transcript levels compared with this treatment without OL (Figure 4C).

Treatment of cells with PAL with or without CER and with or without OL resulted in significant (*P*<0.05) elevation in *Chop* transcript levels compared with control cells, which showed comparable levels with OL treatment. Addition of OL to PAL+CER significantly reduced *Atf4* transcript levels compared with this treatment without OL. Of note are the beneficial effect of OL and the stress effects of CER on *Chop* levels: both high PAL levels and CER and the combination of treatments independently affected *Chop* levels (Figure 4D).

Taken together, the data demonstrate significant elevation in UPR transcript level following acute treatment with PAL, with additional elevation in the presence of CER. These elevations were obliterated in the presence of OL.

### FA acute overload and CER-induced stress; effects on *Pl* transcript levels in primary cells

Primary pancreatic cells treated with PAL (500 µM), with or without CER, demonstrated significant elevation in *Pl* transcript levels compared with control untreated cells, which showed *Pl* transcript levels comparable with those of cells treated with OL and PAL+OL treatments. Thus, OL+PAL treatment significantly reduced *Pl* transcript levels compared with PAL, PAL+CER and CER treatments. CER treatment significantly elevated *Pl* transcript levels when combined with all treatments, including OL, PAL or PAL+OL, as compared with these treatments without CER (Figure 4E).

### FA acute overload and CER-induced stress affect immune transcript levels in primary cells

Treatment with OL, with or without CER, resulted in almost undetectable *Tgf–β* transcript levels comparable with control untreated cells. CER+PAL treatment resulted in high *Tgf–β* transcript levels compared with control. Exposure to high levels of FAs when PAL is part of the combination, with or without CER, resulted in significant elevation in *Tgf–β* transcript levels compared with control untreated cells, and consequently to OL with or without CER. Addition of OL to PAL+CER significantly reduced *Tgf–β* transcript levels compared with this treatment without OL. (Figure 4F).

Aside from treatments with OL, with or without CER, which showed comparable levels to control untreated cells, all other treatments showed significant (*P*<0.05) elevation in *Tnfα* transcript levels compared with control cells. Both CER and PAL independently elevated *Tnfα* transcript levels and had a synergistic effect on *Tnfα* transcript levels. Addition of CER to OL treatment significantly altered *Tnfα* levels; however, the effects seen with both treatments were not different from those in control untreated cells, indicating that OL has a protective effect when combined with CER. Both total fat levels and CER affected *Tnf α* transcript levels; however, this effect was evident only when the type of fat was PAL (Figure 4G).

CER and PAL treatments had both independent and synergistic significant effects on *Il6* transcript levels compared with treatment with OL with or without CER, which showed *Il6* levels similar to or lower than those in untreated control cells. Addition of OL treatment to both CER and PAL treatments resulted in significantly lower *Il6* transcript levels compared with these treatments without OL. Addition of OL to PAL+CER significantly reduced *Il6* transcript levels compared with this treatment without OL. No difference in *Il6* levels was found between OL treatments, with or without CER, indicating that addition of CER to OL treatment did not affect *Il6* transcript levels and emphasizing the protective effect of OL (Figure 4H).

### FA overload and CER-induced stress affected UPR protein expression in exocrine pancreas AR42J cells and in primary acinar cells

**Figure 5:**
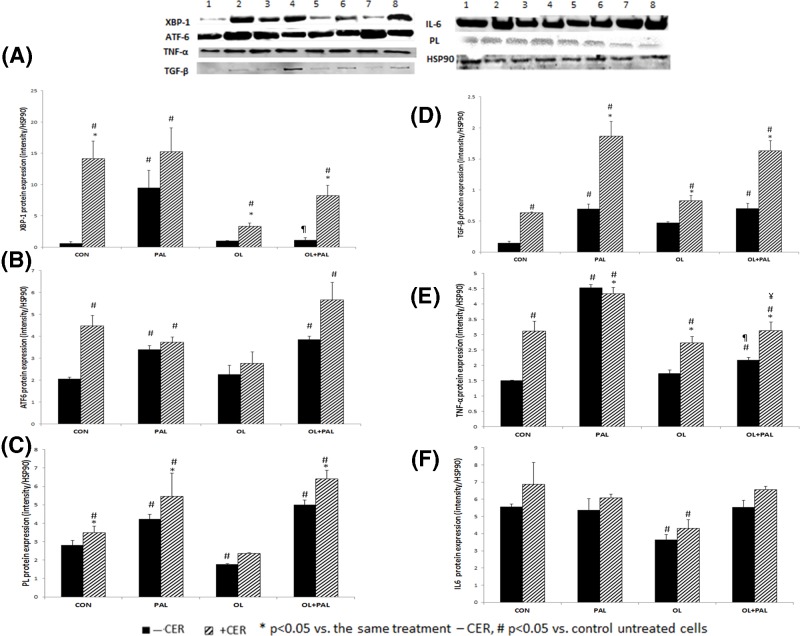
A-G: ER stress, immune and pancreatic enzyme protein levels – in the presence of fatty acids, their combination and CER-induced stress Primary exocrine pancreas acinar cells were isolated and exposed to different FAs, in combination without or with CER (500 µM, - CER – black columns, +CER – hatched column) for 24 h as described in Experimental. Cells were harvested and total protein was isolated and assessed by Western blot. Data were normalized to housekeeping gene HSP90. (**A**) *XBP1* (**B**) *ATF6* (**C**) *PL* (**D**) *TGFβ* (**E**) *TNF*α (**F**) *IL6*. Results are expressed as means ± SEM (*n* = 3). Columns with a superscript are significantly different (*P*<0.05). hash sign # represents significant statistical difference between the treatment versus control; Asterisks * represent significant statistical difference between the treatment and the same treatment with CER. ¶ represent significant statistical difference between OL+PAL versus PAL. ¥ represent significant statistical difference between OL+PAL+CER versus PAL+CER. Upper panel; representative blots of the following treatments; 1 = CON, 2 = CER, 3 = PAL, 4 = PAL+CER, 5 = OL, 6 = OL+CER, 7 = OL+PAL, 8 = OL+PAL+CER.

Treatment of exocrine pancreas primary cells with CER, PAL, PAL+CER or PAL+CER+OL resulted in significant (*P*<0.05) elevation in XBP1 expression compared with cells treated with OL, OL+PAL or untreated control cells, which showed low and comparable levels. Exposure of primary cells to OL+CER treatment resulted in significant mean reduction in XBP1 protein expression compared with cells treated with PAL alone or PAL+ CER+ OL, indicating that PAL alone, and in the presence of CER, elevated ER stress (Figures 5A). The parallel and significant reduction in XBP1 transcript and protein levels following treatments with OL+PAL, OL or OL+CER, to levels of the control untreated cells, and the significant elevation in transcript and protein levels following treatments with CER or PAL+CER compared with other treatments, indicate a transcriptional mechanism by which CER and OL affect XBP1.

Treatment of primary cells with PAL or PAL+OL, with or without CER, resulted in significant elevation in ATF6 protein (full length) expression compared with cells treated with OL without or with CER, which showed levels comparable with those of untreated control cells. Treatment with PAL, PAL+CER or PAL+OL resulted in significant elevation in ATF6 expression compared with cells treated with OL and control cells (Figure 5B).

The parallel changes in ATF6 protein and transcript levels following FA combination, with or without CER, indicate that the amount of fat and CER regulates ATF6 at the transcript level. The type of fat probably regulates ATF6 at the post-transcriptional level or through other complex regulation.

### FA acute overload and CER-induced stress affect PL protein levels in primary cells

Primary acinar cells treated with combination of PAL+OL or PAL alone, with or without CER, showed significant elevation in PL protein levels compared with control untreated cells. In contrast, primary cells treated with OL showed significant decrease compared with control and thus to all other treatments. Treatment of primary cells with PAL+OL with or without CER, or with PAL or OL in combination with CER, resulted in significant elevated PL protein levels compared with OL treatment (Figure 5C). PL transcript and protein levels showed parallel and significant elevation following treatments with OL+PAL+CER and PAL+CER, and a parallel significant reduction following OL+CER treatment, suggesting a transcriptional or post-transcriptional mechanism by which CER-induced stress and OL affect PL.

### FA acute overload and CER-induced stress affect cytokines levels in primary cells

Primary acinar cells treated with PAL without or with OL, and with or without CER, showed significant elevation in TGF-β protein levels compared with control untreated cells and to OL treatment. CER alone or with any of the FAs (OL, PAL or their combination) resulted in significant TGF–β protein elevation compared with untreated and OL-treated cells (Figure 5D). Thus, both CER and PAL treatments have a synergistic effect on TGF–β protein levels. Both amount of fat and type of fat affected TGF–β protein levels. The similar results regarding transcript levels indicate that the regulation of TGF-β by CER and by the amount and type of fat is at the transcript level**.**

Aside from treatment with OL, all other treatments resulted in significant elevation in TNF–α protein levels compared with control. CER alone or combined with FAs (PAL, OL or PAL+OL) resulted in significant elevation in TNF–α protein levels compared with control and OL treatment, which showed comparable levels. CER and PAL treatments independently resulted in the highest TNF–α protein elevation compared with control and OL treatment (Figure 5E). Addition of OL to PAL+CER significantly reduced TNF–α protein levels compared with the same treatment without OL. The similar changes in protein and transcript levels following the different treatments indicate that CER-induced stress and amount and type of fat regulate TNF–α at the transcriptional level.

Similar to TNF–α, apart from cells treated with OL and OL+CER, all other treatments resulted in significant elevation in IL6 levels compared with OL. CER, either alone or combined with PAL or PAL+OL treatments, resulted in significant elevation in IL6 protein levels compared with cells treated with OL and OL+CER (Figure 5F). Parallel changes in IL6 transcript and protein levels following treatment with CER or with the different FAs indicate that the regulation is at the transcriptional level.

### Acute overload of FA, FAs combination and CER induce ER stress: *sXBP* translocation in exocrine pancreas AR42J cells

**Figure 6:**
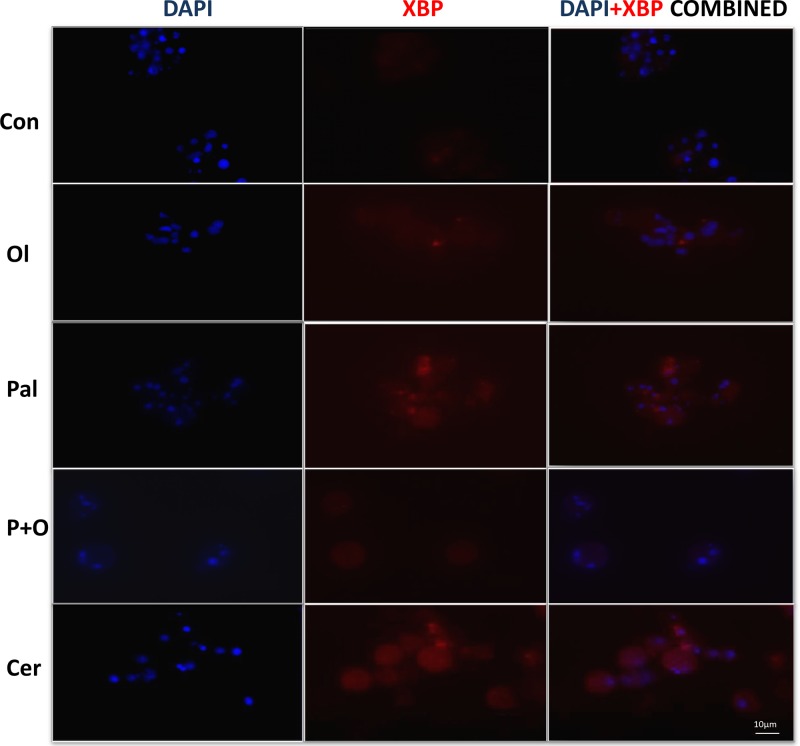
XBP1 trans-localization AR42J cells were treated with CER, OL, PAL and their combination (OL+PAL) as indicated in the Experimental section. XBP1 protein was immune-stained with anti-XBP1 (RED) antibody and the nucleus was stained with DAPI (BLUE). Immunofluorescence was visualized with a LSM 700 Zeiss confocal microscope. XBP1 protein was localized in the cytoplasm under normal and OL treatments but was predominantly translocated into the nuclei by the ER stress induced by both CER and PAL.

We studied the trans-localization of XBP1 following exposure of exocrine pancreas cells (AR42J) to PAL, OL (500 µM), their combination (500 µM for 24 h) and CER by immunocytostaining. The spliced XBP1 is translocated to the nucleus upon ER stress activation. In untreated control cells, XBP1 (red) was localized predominantly to the cytosol (no red overlap with blue nucleus dye), and even there it was barely detectable (red). Similarly, XBP was almost exclusively located in the cytosol following exposure to OL. Following PAL treatment, sXBP1 was translocated to the nucleus and present both in the cytosol and in the nucleus (overlap between red and blue), indicating a pattern of ER activation similar to that induced by CER (Figure 6).

## Discussion

The ER stress UPR is particularly important in maintaining ER homeostasis in professional secretory cells, such as pancreatic acinar cells. Until recently, this mechanism was not studied in depth, particularly in the context of nutrient flow typical to obesity and in relation to stress-induced pathologies of the exocrine pancreas, such as pancreatitis. In a previous study, we have shown that acute exposure to saturated PAL has a deleterious effect on ER stress of exocrine acinar cells, while exposure to mono-unsaturated OL results in a protective effect on ER stress. In the current study, we explored the effects of combination of both FAs on ER and immune stress markers, combined with CER-induced stress, in exocrine *in vitro* AR42J and *ex vivo* primary acinar cells. Our hypothesis was that a combination of FAs will reduce the ER stress UPR, particularly in the presence of CER-induced stress, mimicking a cellular state of pancreatitis. Our data validate our hypothesis and show that exposure to a combination of monounsaturated and saturated FAs protect exocrine pancreatic cells from ER stress response induced by saturated FAs.

Acute exposure (24 h) to PAL resulted in a significant elevation in ER stress in a concentration-dependent gradual manner; higher levels (500 µM), typical and reflecting blood levels in human obesity, resulted in higher elevation in ER stress markers compared with exposure to lower levels (250 µM), as indicated by UPR transcript and protein levels of ATF4, ATF6 and XBP1 and in XBP1 splicing levels. Exposure to OL resulted in reduction in these ER stress markers to levels comparable to those in control untreated cells. Similar parallel changes in transcript and protein levels following exposure to specific doses and types of FAs indicate that the effects of exposure to FAs are probably at the pre-transcriptional or transcriptional level.

ER stress plays a significant role in the ability of cells to cope with nutrient flow and its fluctuations. Nutrients, in particular saturated fats, are known to induce ER stress. Similar results to ours were found in stem cells and mesenchymal pre-adipocytes, where exposure to PAL led to increase in ER stress, whereas exposure to OL reduced stress levels as observed by XBP1 splicing. In addition, exposure of HepG2 and Huh7 human liver cells to PAL led to an increase in UPR-related ATF6 transcript and protein levels [25]. Also, mice fed a HFD as well as ob/ob mice show activation of UPR in both adipose tissue and liver [15,16].

The protective effects of mono-unsaturated acids on the toxifying exposure to saturated FAs have been studied in different cells; however, not in exocrine pancreas stress and especially not in relation to ER stress [4]. Our results show that compared with exposure to high levels of PAL, acute exposure to the combination of PAL and OL (1:1) resulted in significant reduction in the toxic effect of PAL, as indicated by reduced levels of the UPR markers ATF4, CHOP and XBP1 at the transcript and protein levels, as well as XBP1 splicing and sXBP1 translocation. Activation of ATF4 and CHOP may result in apoptosis; thus, the protective effect of OL ameliorates the apoptotic path. XBP1 belongs to a bZIP family of transcription factors. XBP1 translocation from the cytosol to the nucleus is one of the clear fingerprints of UPR response, initiating and activating the full UPR response by transcriptional induction of genes involved in the response.

Similar to our results, research done in β-cells (INS-1s) found that a combination of PAL:OL protected cells from apoptosis induced by PAL [38,39]. Additionally, in our previous work we have shown that long exposure to PAL induces lipotoxic apoptotic effects in AR42J exocrine cells [24]. The protective effect of OL is probably distinct and unique to OL, as exposure to a combination of PAL and OL did not alter ATF6 transcript and protein levels compared with PAL alone. This selective phenomenon was also previously reported in HepG2 hepatocytes [40].

Obesity and HFD are linked to elevated risk and aggravation of pancreatic pathologies: pancreatitis and pancreatic cancer [41,42]. Furthermore, ER stress pathways were found to be closely linked to mechanisms involved in immunity and inflammation [4]. Our data indicate that acute exposure to high levels of PAL, typical of obesity, results in a pro-inflammatory state in primary exocrine pancreas cells, expressed by significant elevation in TNF-α, IL-6 and TGF-β transcript and protein levels, compared with OL exposure and control untreated cells, which showed low and comparable levels of inflammatory cytokines. The parallel change in transcript and protein levels indicates a pre-transcriptional or transcriptional mechanism. Furthermore, exposure to high OL levels alone resulted in significant reduction in TNF-α transcript, reinforcing the protective effect of OL. Similar to our study, studies in β-cells (INE-1E) and keratinocytes (HaCaT) showed that PAL exposure induces production of pro-inflammatory cytokines (TNF-α, IL-6 and TGF-β) [43,44]. Also, studies, in β-cells showed that OL exposure reduces the effect of TNF-α on insulin resistance [45]; and in human endothelial cells, exposure to OL (unlike other FAs) down-regulated pro-inflammatory markers, including TNF-α [46]. Although the mechanism of these effects is not known, preliminary data indicate possible involvement of activation of NF-kB, calcium signaling and the AMPK pathway.

The main role of exocrine pancreatic cells is synthesis and secretion of digestive enzymes [47,48]. Pancreatic lipase (PL), one of the main pancreatic digestive enzymes, responds selectively to dietary fat and serves as one of the main diagnostic markers for pancreatitis [49]. Exposure of exocrine pancreatic primary cells to normal (250 µM) and high (500 µM) levels of PAL resulted in significant elevation in PL protein and transcript levels, compared with exposure to OL and control. Exposure to combination of PAL:OL (1:1) resulted in significant reduction in PL levels compared with exposure to PAL alone.

The etiology and risk factors of pancreatitis (acute or chronic) are not fully known, and involve several environmental factors, including lifestyle and diet. Obesity in general and abdominal obesity in particular are linked to the severity of the systemic inflammatory response in acute pancreatitis [50]. In several pancreatitis models, there is evidence for a role of ER stress, expressed by alterations in gene expression and in ER structure in early development of pancreatitis [47,51]. In fact, pancreatitis induced in *LepDb* and *LepOb* genetically obese mice is more severe than in lean mice [49]. Furthermore, FA profiles in inflamed rat pancreas show elevation in PAL concentration [49,50]. Moreover, several studies related to different pancreatic pathologies found that FFAs aggravate pancreatitis. The type of FAs in this context varies between pancreatic pathological state and type. For instance, it has been shown that mainly mono-unsaturated FAs play a role in the aggravation of acute edematous pancreatitis, indicated by elevated unsaturated FAs in plasma of pancreatitis patients and induction of necrosis in several pathways [52–55]. Similar to our results, Zeng et al. showed that induction of pancreatitis by palmitic acid *in vitro* in acinar cells and *in vivo* aggravates pancreatitis induced by CCK8 and CER, respectively, as shown by ER transcripts, amylase section and calcium accumulation [56]. Our results further suggest a possible link between FFA and pancreatitis, though evidently further *in vivo* and *ex vivo* studies are essential to confirm this link in animal models and humans. Furthermore, the data suggest that different cellular organs and different FAs act at different points throughout the pancreatic pathology process.

We now show that not only exposure to high levels of saturated FAs induces ER stress and inflammation markers, but that in a state of CER-induced stress, exposure to saturated FAs further aggravates the stress: exposure to high levels of PAL in parallel to induction of CER-induced stress further aggravates the stress and inflammation markers. We demonstrate that in the presence of CER-induced stress, exposure to OL protects exocrine pancreas cells by reducing ER stress, as expressed by significant reduction in CHOP, XBP, ATF4 and ATF6 and the cytokines TGF-β, TNF-α and IL-6. Additionally, PL is also down-regulated. In pancreatitis, a combination of both PAL and OL (1:1) significantly reduced ER stress. Interestingly, without induction of CER, the protective effect of OL was not mediated through ATF6. A previous publication found, similar to our study, that ATF6 or PERK phosphorylation and IRE1 are not reliable ER markers, suggesting that detection of downstream protein targets of ER stress, such as CHOP, XBP1 and ATF4, represent a more robust approach for detecting activation of the UPR. The combination of FAs did not alter the inflammatory cytokines [57].

It is noteworthy that our research has, as mechanism-focused research at the cell level often does, some limitations. Although we studied standard exocrine pancreas models (AR42J cells and primary exocrine pancreas cells), they might not represent the full *in vivo*, whole body interaction, especially in severe diseases, such as pancreatitis. Additionally, although PAL and OL are the more abundant FFAs, other FFA profiles exist in other physiological situations.

In conclusion, previous studies have demonstrated that obesity and dietary FA profile significantly affect ER stress levels in the exocrine pancreas and pancreatitis. We have previously shown that exocrine pancreatic cells store fat. Using different models of exocrine pancreas cells (*in vitro* AR42J and *ex vivo* primary exocrine pancreas cells) we now demonstrate that saturated FA (PAL) has a poisonous effect through exacerbation of ER stress and aggravation of pancreatic stress markers. In contrast, exposure to monounsaturated FA (OL), even at high levels, does not induce ER stress and has a protective anti-stress and anti-inflammatory effect. Combination of both FAs reduced the PAL-induced ER stress in both normal and CER-induced stress states. However, this protective effect did not affect inflammation markers. Further studies testing the possible relevance of these findings to pancreatitis and their validity in *in vivo* and other *ex vivo* systems in animal models and humans will determine possible applicability of the findings to clinical nutritional practice.

## Abbreviations

ATF6: activating transcription factor-6
CHOP: CCAAT-enhancer-binding protein homologous protein
ER: endoplasmic reticulum
FFA: free fatty acid
HFD: high fat diet
IRE1: Inositol-requiring enzyme-1
OL: oleic acid
PERK: protein kinase RNA-activated-like ER kinase
PL: pancreatic lipase
QPCR: quantitative PCR
UPR: unfolded protein response
XBP1: X-box binding protein 1
